# The electron density: a fidelity witness for quantum computation†

**DOI:** 10.1039/d3sc05269a

**Published:** 2023-12-20

**Authors:** Mårten Skogh, Werner Dobrautz, Phalgun Lolur, Christopher Warren, Janka Biznárová, Amr Osman, Giovanna Tancredi, Jonas Bylander, Martin Rahm

**Affiliations:** a Department of Chemistry and Chemical Engineering, Chalmers University of Technology Gothenburg Sweden martin.rahm@chalmers.se; b Data Science & Modelling, Pharmaceutical Science, R&D, AstraZeneca Gothenburg Sweden; c Department of Microtechnology and Nanoscience MC2, Chalmers University of Technology Gothenburg Sweden

## Abstract

There is currently no combination of quantum hardware and algorithms that can provide an advantage over conventional calculations of molecules or materials. However, if or when such a point is reached, new strategies will be needed to verify predictions made using quantum devices. We propose that the electron density, obtained through experimental or computational means, can serve as a robust benchmark for validating the accuracy of quantum computation of chemistry. An initial exploration into topological features of electron densities, facilitated by quantum computation, is presented here as a proof of concept. Additionally, we examine the effects of constraining and symmetrizing measured one-particle reduced density matrices on noise-driven errors in the electron density distribution. We emphasize the potential benefits and future need for high-quality electron densities derived from diffraction experiments for validating classically intractable quantum computations of materials.

## Abstract 2

We propose that the electron density, obtained through experimental or computational means, can serve as a robust benchmark for validating the accuracy of quantum computation of chemistry.

## Graphical abstract

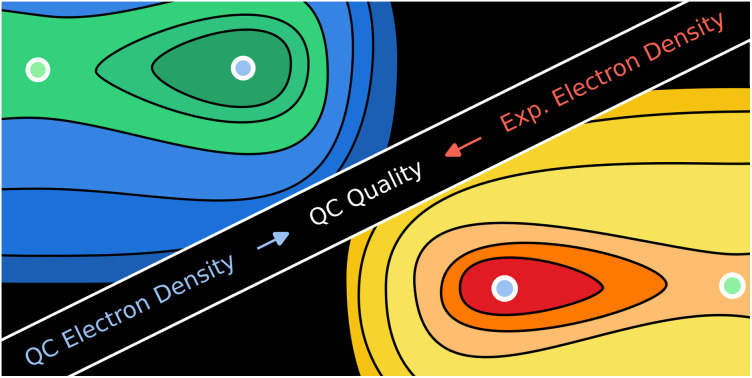

## Introduction

In this study, we demonstrate the evaluation of electron densities of molecules using quantum computation. We also suggest that the electron density can be a potent validation tool of future quantum calculations, which may prove intractable to solve with conventional quantum chemistry. The study of electron densities is central to several fields of chemistry, physics, and materials science. The Hohenberg–Kohn theorem stipulates that the electron density uniquely defines ground state properties of electronic systems.^1^ Through the Hellmann–Feynman theorem,^2^ electron densities provide information on the forces acting within molecules.^3,4^ As one of the most information-rich observables in physical sciences,^5–10^ the density lays the foundation for density functional theory (DFT), a formalism for predicting properties of many-electron systems.^11^ As experiments are the arbiter of truth, the buck often stops with the electron density.

Electron densities can, importantly, be reconstructed from the refinement of X-ray diffraction and scattering data,^9^ using, *e.g.*, multipolar models,^5–8,10^ X-ray constrained wave functions,^12^ or the maximum entropy method.^13^ One motivation for our work is that experimentally determined electron densities can prove useful for testing the accuracy of future quantum computations of materials; simulations of which may be unfeasible with conventional computers. Today, it is often preferable, cheaper, and faster to obtain information on electron distribution through conventional quantum mechanical calculations, *e.g.*, by solving the Schrödinger equation at some level of approximation.^14^ To obtain highly accurate computational results (energies, densities, or other properties), *ab initio* quantum mechanical methods are the most reliable. Unfortunately, the electronic structure problem scales exponentially with system size.^2^

In 1980 and 1981, Benioff,^15^ Manin^16^ and Feynman^17^ pointed out that quantum computers may offer a way forward, enabling larger and more reliable simulations of quantum systems.^18^ In recent years, several quantum devices have been developed and applied to compute energies of small molecules (see *e.g.*,^19–21^). No such calculation has, however, yet exceeded the accuracy or speed of a conventional quantum chemical calculation. So, just how far away are we from useful quantum computation of chemistry? To quantifiably answer that question, it is necessary to perform benchmarking and validate the results of quantum computation in some manner. Thus far, such efforts have almost exclusively focused on a comparison against total energies obtained using state-of-the-art (and near exact) quantum chemistry.^22–25^ However, whereas accurate energies for smaller molecules are available, such comparisons will not be possible if future quantum calculations of more complex systems are made possible. We emphasize at the onset that whether the crossing of such a technological threshold is made in the current noisy intermediate-scale quantum (NISQ) era or if it will require full fault tolerance quantum devices does not affect our main message or conclusions. Here, we suggest that the subtle variability of electron densities, accessible either computationally or experimentally, can act as a potent benchmark^26^ for the quantum computation of materials. We draw the colloquial analogy to fidelity witnesses in the title,^27^ by which we mean experimentally accessible observables whose values (here in terms of topological features) help to quantify the fidelity of a quantum calculation.

In what follows, we demonstrate calculations of electron densities (and their topological features) of molecular hydrogen (H_2_) and lithium hydride (LiH) using quantum computers. These molecules are archetypical examples of fundamentally different chemical bonds (covalent and ionic). Simulation of quantum hardware is also employed to derive the electron density of larger molecules, the lithium dimer (Li_2_) and hydrogen cyanide (HCN). The quantum volume^28^ and noise level of the devices we use are insufficient to demonstrate any advantage over classical implementations (details of the hardware are provided in the Methods section and ESI†).^29^ However, they suffice for our goal – a first proof-of-principle evaluation of electron densities using quantum devices. By comparing aspects of the electron density topology in these molecules, we showcase a different way to benchmark the quality of quantum hardware calculations of chemistry. To do so, we make use of the quantum theory of atoms in molecules (QTAIM),^30–32^ a well-established framework for performing topological analysis of electron densities, which can provide clues into atomic properties,^7^ chemical bonding,^8,10^ lattice energies,^33^ chemical bond strengths, and reactivity.^34–37^

## Topological analysis of electron densities

Within the Born–Oppenheimer formalism, the average one-electron density for a system of *N* electrons can be expressed as^38^1
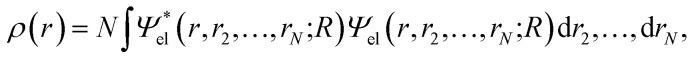
where *r*, *R*, and *Ψ*_el_ denote the electronic and nuclear coordinates and the electronic wave function, respectively. The electronic density is a real-valued scalar field, lending itself to topological analysis and the extraction of topological features *f*_*χ*_. One way to analyze such topology is to study critical points (CPs), *i.e.*, locations in the density where the gradient vanishes, ∇*ρ* = 0, or is undefined.^39^ The critical points form a concise set of features that yield insight into the molecular structure.

To characterize and distinguish between critical points, we make use of the Hessian and its trace, the Laplacian (or curvature) of the density,2
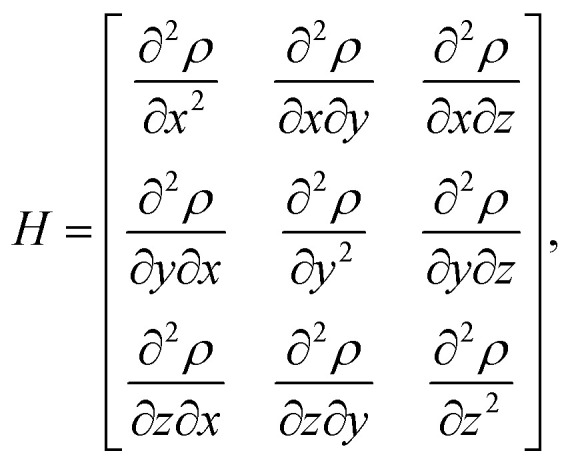
3
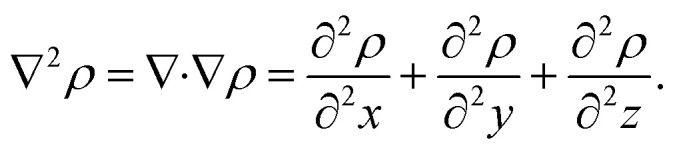


For our analysis and discussion, it suffices to distinguish between critical points using so-called signatures, *κ*, defined as the sum of the signs of the three eigenvalues of the Hessian.^40^ The critical points in molecules we will analyze are of two forms: *κ* = −3 indicates local maxima, such as positions of nuclei, where all curvatures are negative. A non-nuclear attractor (NNA) is a rare example of a CP located at off-nuclei positions. We will discuss one NNA in Li_2_. In contrast, *κ* = −1 corresponds to saddle points in the electron density, *i.e.*, positions where the curvature in one direction is positive. The latter topological feature is commonly referred to as bond-critical points (BCPs) because they are often (but not always) found between neighboring atoms that are chemically bound. A BCP is a point of lowest electron density along a path of highest electron density; the lowest point along a ridge connecting maxima. Because the density uniquely defines them, BCPs constitute suitable points of comparison between levels of theory and experiment. BCPs are furthermore useful for characterizing bonds in different ways.^41^ For example, the sign of the Laplacian (eqn (3)) at a BCP is an indication of local depletion (if positive) or concentration (if negative) of the electron density relative to its surroundings. A negative sign of ∇^2^*ρ* indicates a covalent bond, while a positive sign hints at an ionic (or closed-shell) type of interaction.

**Fig. 1:**
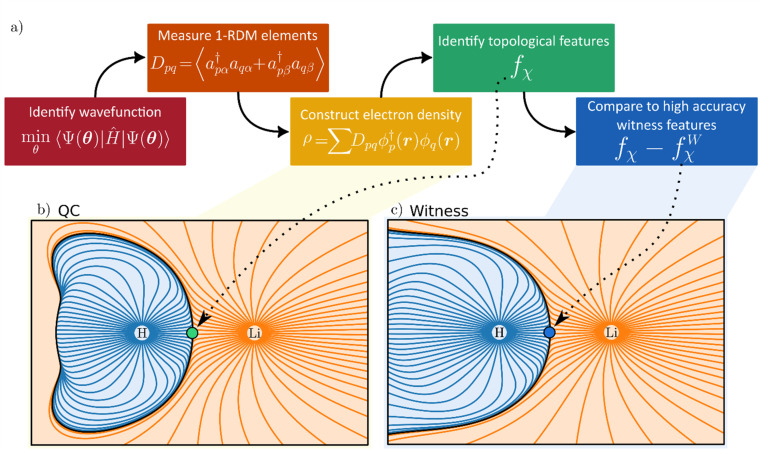
(a) Five-step procedure for electron density-based witness estimation. A wavefunction is initially converged using a quantum algorithm. The corresponding one-particle reduced density matrix (1-RDM, ***D***) is subsequently measured from the converged wavefunction. The electron density, *ρ*, is constructed from the measured 1-RDM. Topological analysis of the constructed electron density generates topological features *f*_*χ*_, such as critical points. Finally, the quality of the quantum calculation can be evaluated by comparing topological features to a known (possibly experimentally determined) witness. (b) Gradient field lines of the electron density and atomic basins in LiH derived from quantum computation (QC). (c) Gradient field lines and atomic basins for the near-exact witness, derived from conventional quantum chemistry calculations at the CCSD/aug-cc-pVTZ level of theory. Green and blue circles indicate BCPs in data from the quantum calculation and witness, respectively.

Topological analysis of electron densities (*viz.* QTAIM) also offers a way of defining atoms within molecules.^40^ Within QTAIM, atoms are identified with basins (Fig. 1b and c), non-overlapping regions of space within which all gradient trajectories of the electron density terminate at the same local maximum (*i.e.*, a *κ* = −3 critical point). Note, therefore, that basins need not strictly be centered around nuclei but can be associated with NNAs. Neighboring basins are separated by zero-flux surfaces where ∇*ρ*(***r***)·***n*** = 0, and where ***n*** is the normal to the surface at ***r***. We will use such basins to ascribe partial charge to atoms inside molecules, and we suggest such measures, along with *ρ* and ∇^2^*ρ* at BCPs, as examples of potent density-based quantum computational fidelity witnesses.

## Quantum computation of electron densities

For quantum computation, the electronic structure problem is conveniently expressed within second quantization.^42^ Within this formalism, the electron density can be defined as4
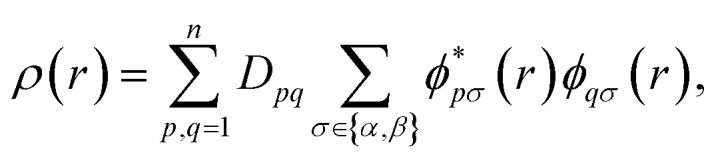
where 
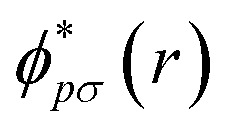
 and *ϕ*_*qσ*_(*r*) correspond to the *n* spin orbitals and *p* and *σ* denote spatial and spin indices, respectively. *D*_*pq*_ is an entry in the one-particle reduced density matrix (1-RDM).^43^ In the following, we use *p* and *q* as spatial variables and *σ* and *τ* to index spin, in turn, denoted as *α* or *β* (*i.e.*, *σ*,*τ* ∈ {*α*,*β*}). The 1-RDM can be expressed as,5
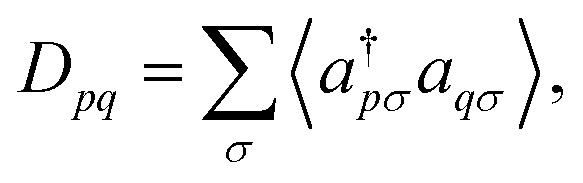
where,

6 〈*a*^†^_*pσ*_*a*_*qσ*_〉 = 〈*Ψ*|*a*^†^_*pσ*_*a*_*qσ*_|*Ψ*〉.

In eqn (5), *a*^†^_*pσ*_ and *a*_*qσ*_ are the fermionic ladder (creation and annihilation) operators of electrons in spin orbital *ϕ*_*qσ*_(*r*) while |*Ψ*〉 represents the wave function, defined as a linear combination of Slater determinants, |*ψ*_*i*_〉,7
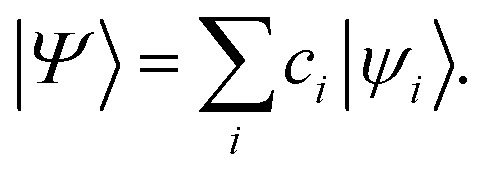


In this work, 1-RDMs are constructed following measurements of parametrized quantum circuits (ansätze), representing the ground state of molecules of interest. Measurement of the 1-RDM scales as *O*(*n*^2^), rendering a density-based fidelity witness approach computationally efficient. We will return to discuss the sensitivity of *D*_*pq*_ to noise on quantum devices and how the outcome of calculations can be affected by enforcing physically motivated constraints on the 1-RDM.

Several algorithms^18,44,45^ can be utilized to prepare the ground state solution, |*Ψ*_GS_〉, of a molecule by encoding the fermionic chemistry problem^18,46,47^ onto a quantum computer. We rely on the variational quantum eigensolver^48^ (VQE) algorithm in this study, as it has been thoroughly used with the current generation of quantum devices. The VQE algorithm, outlined elsewhere,^21,22^ leverages both quantum and classical computation to iteratively optimize a parameterized quantum circuit *U*(***θ***). It minimizes the expectation value of the Hamiltonian, 
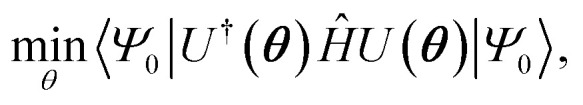
 where |*Ψ*_0_〉 is the initial reference state, usually the Hartree–Fock configuration. The ground state problem can be reduced to the electronic Hamiltonian, defined in second quantization as8
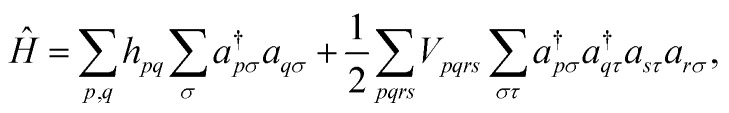
where *h*_*pq*_ and *V*_*pqrs*_ are the one- and two-electron integrals. We stress that our specific choices of algorithms and encoding procedures are not essential for the general case of calculating electron densities with a quantum computer. What is necessary is (a) identification of a state of interest (in our case, the ground state) by some quantum algorithm or quantum simulation,^49^ and (b) reconstruction of the 1-RDM through measurements of 〈*a*^†^_*p*_*a*_*q*_〉, following eqn (5).

## Effects of noise

Noise is perhaps the single most defining characteristic of current quantum computing and the NISQ era.^29^ NISQ algorithms are often hybrid in nature, dividing the computational load between both quantum and conventional hardware. In our case, the orbitals *ϕ*_*pσ*_(*r*) and *ϕ*_*qτ*_(*r*) of eqn (4) are precisely known functions represented on a conventional computer and assumed errorless. The quantum computer, in turn, stores the orbital occupations and phase. It is in the quantum computer where noise enters as uncertainty and measurement errors. As our interest lies with the 1-RDM, we will focus on the specific effects of noise on the elements *D*_*pq*_.

By defining elementwise errors as *ε*_*pq*_ = *D*_*pq*_ − *D*^NF^_*pq*_, where *D*^NF^_*pq*_ is a noise- and error-free reference value, we can divide the effects of noise on the off-diagonal elements into two categories: diagonally symmetric (*ε*_*ij*_ = *ε*_*ji*_) and asymmetric (*ε*_*ij*_ ≠ *ε*_*ji*_) errors.

To see how different kinds of noise may affect off-diagonal elements, we first look at a Jordan–Wigner mapping of our fermionic creation and annihilation operators:

9 *a*^†^_*pσ*_ = (*X*_*k*_ − *iY*_*k*_) ⊗ *Z*^*k*→^

10 *a*_*pσ*_ = (*X*_*k*_ + *iY*_*k*_) ⊗ *Z*^*k*→^.

We here use *k* to index our qubits, where each qubit *k* maps to a unique spin orbital, *ϕ*_*pσ*_. *X* and *Y* represent the corresponding Pauli gates, whereas *Z*^*k*→^ is the application of Pauli *Z* gates to all qubits *k* − 1, *k* − 2, …, 1. Note that eqn (9) and (10) are identical except for the sign (phase) of the *Y*-gate. In practice, this similarity means that the measurements of 〈*a*^†^_*pσ*_*a*_*qτ*_〉 and 〈*a*^†^_*qτ*_*a*_*qσ*_〉 will both perform the same measurements of Pauli strings *X*_*k*_ ⊗ *Z*^*k*→^ and *Y*_*k*_ ⊗ *Z*^*k*→^, with the phase introduced as a classical coefficient. Any measured diagonal asymmetries in the 1-RDM should, therefore, be solely due to insufficient sampling of the noisy state. Thus, provided independent measurements, and time-independent noise for a given set of circuit parameters, ***θ***, we can expect to sample the same noisy quantum state *P*(***θ***) with every measurement. Here, we use independent measurements to mean single evaluations of the same quantum circuit that are not affected by previous evaluations. We phrase the expectation value in density matrix representation (not to be confused with a reduced density matrix), where *P*(***θ***) is the parameterized density matrix. In this representation, the expectation value of an operator *Ô* is given as 〈*Ô*〉 = Tr[*PÔ*]. In other words, provided that the above assumptions hold, any pair of real-valued off-diagonal elements must be equal, Tr[*P*(***θ***)*a*^†^_*i*_*a*_*j*_] = Tr[*P*(***θ***)*a*^†^_*j*_*a*_*i*_].

While noise is an unavoidable part of contemporary quantum computing, symmetries and properties in the studied system can often be used to gauge and combat errors. As such, it is of interest to study how the 1-RDM and derived properties thereof are affected by noise. And conversely, how enforcing known symmetries and properties of the 1-RDM mitigates the effect of noise. We have opted to study two important properties of the 1-RDM: its hermiticity, 
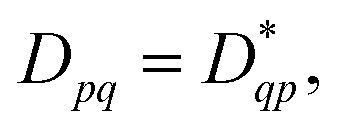
 and that its trace equals the number of electrons of the studied molecular system,^52^ Tr(***D***) = *n*_el_.^50^

Because the 1-RDM is Hermitian, we can, assuming real-valued entries, enforce the desired transpose (*D*_*pq*_ = *D*_*qp*_) symmetry on the measured 1-RDMs by averaging the corresponding off-diagonal elements as11
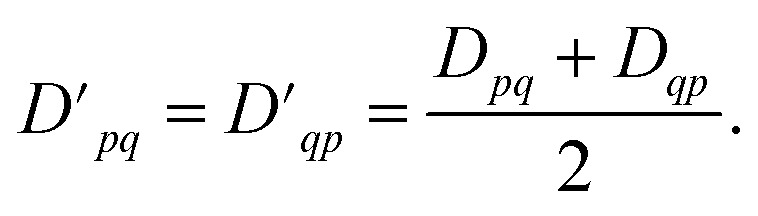


We also ensure particle conservation by normalizing the sum of all diagonal elements, Tr(***D***), to equal the total number of electrons, *n*_el_,12
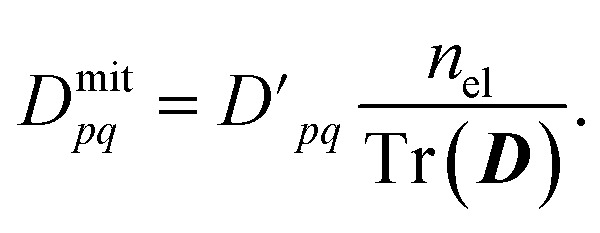


Diagonal elements of the density matrix can be measured directly on quantum hardware, while off-diagonal elements require rotations to the measurement basis. While these basis rotations technically introduce more noise to the off-diagonal elements, these effects will be dominated by the noise incurred by preparing the (approximate) ground state wavefunction. This is because the circuit ansatz to prepare the ground state contains many more gates (including noisier 2-qubit gates) compared to the measurement basis rotations. Thus, diagonal and off-diagonal elements are expected to be affected by similar degrees of noise and are subject to the same rescaling. We refer to the combined result of eqn (11) and (12) as a noise-mitigated 1-RDM. Our mitigation approach is not unique in relying on 1- or 2-RDMs to reduce errors, and similar techniques have been implemented by others.^51–53^ Thus, we emphasize that our aim is not to propose a novel mitigation strategy but rather to study its effect on a noisy 1-RDM and the resulting topology of the electron density. As our focus lies in evaluating topological qualities of the electron densities in the presence of noise and errors, we have opted for a conceptually straightforward mitigation strategy. As will be noted in the Results and discussion section, some noise effects on the measured number of electrons can already be avoided by using certain fermion-to-qubit encodings and qubit tapering. In particular, the use of parity encoding^46^ allows us to preserve the parity of the number of particles of each spin species (*α* and *β*). For the case of lone *α* and *β* electrons, such as the singlet H_2_, the parity conservation coincides with particle conservation. Consequently, performing the parity transformation in such cases will effectively protect against any error in the particle or projected spin numbers.

## Results and discussion

To evaluate the viability of the electron density's topological properties as a form of fidelity witness, we perform quantum chemistry calculations on both quantum and conventional computers. The quantum calculations rely on hardware-efficient ansätze^54^ and minimal or small basis sets to expand the molecular wavefunction (see the Methods section). We use high-quality electron densities from conventional coupled cluster calculations at the CCSD/aug-cc-pVTZ level of theory as reference data. We note that reference densities can, in principle (up to a feasible limit), be obtained in many ways, including other costly *ab initio* methods, more affordable DFT functionals, diffraction data, or even wavefunction-fitting experiments.^55^

Central to our work are analyses of the significant contribution of noise on the quality of electron densities obtained with current quantum calculations. To that end, we not only compare with noiseless quantum simulations and near-exact conventional calculations, but we also implement and evaluate the efficacy of the above-described mitigation strategy for topological properties. To make our test set feasible on available quantum hardware, we rely on small basis sets (STO-3G for H_2_, LiH and Li_2_, and 6-31G for HCN). A frozen (1s) core approximation is used for atoms heavier than H, together with relatively small active spaces: H_2_ (2,2), LiH (2,3), Li_2_ (2,4), and HCN (4,4). The canonical molecular orbitals used to define these active spaces are provided in the ESI (Fig. S5–S8†). Two kinds of encodings are used to map the fermionic spin orbitals to qubits: parity encoding with two-qubit reduction for H_2_ and LiH, and Jordan–Wigner encoding for Li_2_ and HCN. While quantum results from H_2_ and LiH are obtained from real quantum hardware, results for Li_2_ and HCN are from simulations of quantum hardware that include a depolarizing noise model. Specifics of the noise model and further computational details are available in the Methods section and the ESI.†

**Fig. 2:**
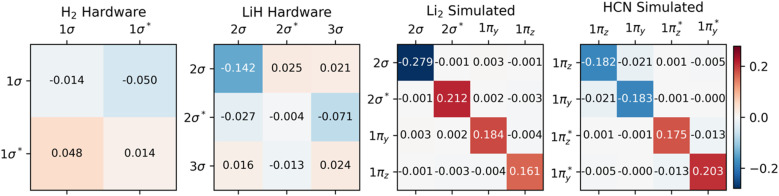
Errors in 1-RDMs, comparing quantum devices (hardware and simulated) to exact (statevector) calculations. Color and intensity indicate the sign and magnitude of the errors, respectively. Hardware data (for H_2_ and LiH) demonstrate higher asymmetry than simulations (of Li_2_, HCN) with depolarizing noise models. Only 1-RDM elements that correspond to the active space are shown. All bonds are aligned with the *x*-axis.

Fig. 2 demonstrates that significant errors can be present in both the diagonal and off-diagonal elements of the 1-RDMs. Similar conclusions have been drawn by Arute *et al.*^52^ and Smart *et al.*^56^ Note that an error along the diagonal is especially detrimental, as this – unless there is fortuitous error cancellation – can result in an incorrect (and unphysical) total number of electrons (Table 1). Even when the number of electrons is conserved by means of the encoding method, as in the case of H_2_, errors are still apparent along the diagonal of the 1-RDM. Table 1 summarizes how over- and underestimation of the true number of electrons is (a) present for larger molecules and (b) can be effectively corrected by rescaling the 1-RDM.

**Table 1:** Number of electrons obtained from the trace of the 1-RDMs. For H_2_, the noisy calculation retains all electrons due to parity encoding and qubit tapering. Our calculations of larger molecules deviate noticeably from the correct number of electrons due to noise, effects that can be corrected by error mitigation. Estimated error bounds are given as a standard deviation based on the number of samples used in the measurements. The electron number for H_2_ is not affected by the number of samples

Molecule	Number of electrons		
	Noise-free	Noisy	Mitigated
H_2_	2.00	2.00^a^	2.00
LiH	4.00	3.88^b^ ± 0.011	4.00
Li_2_	6.00	6.28^c^ ± 0.003	6.00
HCN	14.00	14.01^c^ ± 0.003	14.00

a Chalmers Särimner device.

b ibmq_quito device.

c Simulation using a depolarizing noise model.

We want to reiterate that errors in the off-diagonal elements come in two flavors: symmetric and asymmetric with respect to the diagonal. Note that the latter type of error only arises in the data from real quantum devices. The found asymmetry agrees well with the reduced number of measurement samples used in these calculations relative to the 10^6^ samples applied in our quantum simulations. Physical hardware can also experience noise levels fluctuating over time, breaking our previous assumption of time-invariant noise.

Moving to topological analysis of our electron densities, we look first at atomic partial charges (Table 2). The partial charges attributed to each topological atom are evaluated by partitioning space into atomic QTAIM basins (Fig. 1b and c). Table 2 shows atomic partial charges derived from such topological atoms and how noise can affect the quantification of this important chemical concept. Also shown in Table 2 are the results following the application of our error mitigation strategy of rescaling and symmetrizing the 1-RDM. Whereas the latter approach generally improves results, its occasional failures can impart valuable lessons on how to improve chemically informed error mitigation.

**Table 2:** Atomic partial charges derived from a topological QTAIM analysis of the electron density. Rescaling and symmetrization of 1-RDM results generally improve computed partial charges, with the clear exceptions of the NNA of Li_2_

Molecule	Atom	Partial atomic charge		
		Noise-free	Noisy	Mitigated
H_2_	H	0.00	0.00^a^	0.00
	H	0.00	0.00^a^	0.00
LiH	Li	0.84	0.85^b^	0.85
	H	−0.84	−0.73^b^	−0.85
Li_2_	Li	0.32	0.07^c^	0.16
	NNA	−0.65	−0.42^c^	−0.32
	Li	0.32	0.07^c^	0.16
HCN	C	0.89	0.74^c^	0.75
	N	−1.07	−0.94^c^	−0.93
	H	0.18	0.18^c^	0.18

a Chalmers Särimner device.

b ibmq_quito device.

c Simulation using a depolarizing noise model.

**Fig. 3:**
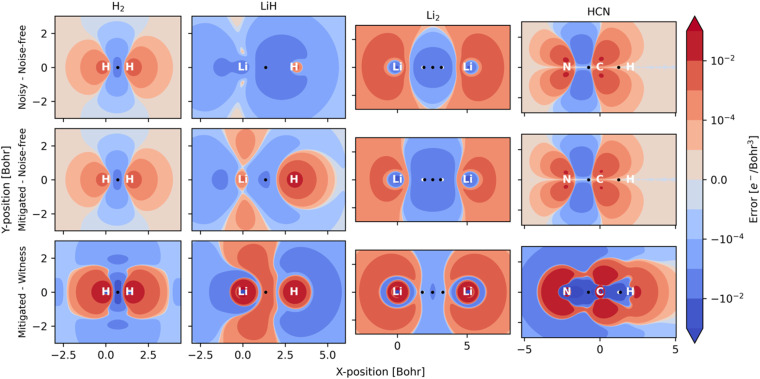
Electron density difference between noisy and noise-free (top), mitigated and noise-free (middle), and between mitigated and witness (bottom) results. The increased occupation of anti-bonding orbitals due to noise can be seen in all studied systems, resulting in a decreased electron density between nuclei.

One case of failure of our mitigation strategy lies with the NNA of Li_2_, a rare (but well-known) feature in the electron density where a local maximum is present between the two nuclei (Fig. 3). One explanation for the reduced accuracy of the NNA's partial charge is a substantial over-occupation due to noise of the valence π orbitals and the 2σ* orbital (Fig. S4†), all of which have but a small overlap with the NNA basin. In contrast, the valence 2σ orbital exhibits a significant reduction in population due to noise and overlaps prominently with the NNA basin. In other words, it is the incorrect relative filling due to noise of the 1π and 2σ* orbitals over the 2σ that effectively removes electrons from the inter-nuclear NNA basin in favor of the nuclear basins. Because the rescaling aspect of our error mitigation strategy (*viz.*eqn (12)) acts on all orbitals proportionally to their occupation, the 2σ is scaled down to a larger extent, further exaggerating the error in this case.

One example where our approach to error mitigation does little to correct topological features is HCN. The main reason for this is that the used active space is small and only includes the two bonding π and two anti-bonding π* orbitals. In contrast to our example of Li_2_, the π orbitals in HCN are polarized due to the difference in electronegativity of N and C. The π* orbitals are more pronounced on C and, consequently, have a larger overlap with the identified C basin. In contrast, the π orbitals have a larger N contribution and overlap slightly more with the N basin. Because the net effect of noise is an overpopulation of the antibonding π* orbitals (Fig. S4†), the charge distribution is slightly skewed in favor of C (Table 2). A second reason for the negligible effect of our mitigation scheme in the case of HCN is that our calculations are based on sufficiently many, 10^6^, simulated measurements, which effectively removes asymmetric errors in the 1-RDM. The main advantage of our error mitigation approach in this example is ensuring the correct number of electrons (Table 1).

Fig. 3 illustrates the effect of noise as electron density difference maps in planes across the different molecules, while Table 3 quantifies noise (and mitigation) at selected critical points in the density. Because our calculations rely on a hybrid-quantum-classical algorithm, where orbitals are handled on a classical computer, electron densities are always constrained by the symmetries inherent to those orbitals. Such symmetries can also protect against noise along certain mirror planes. For example, Table 3 shows how the electron density at BCPs reported for HCN are all invariant to noise. This robustness is not general but a consequence of the small (4e, 4o) active space used. Because the π and π*orbitals have nodal planes along the bond axis, it is only in the Laplacian of *ρ*(***r***) that one can distinguish between noisy, mitigated, and noise-free results for these points within HCN. For Li_2_, the situation is reversed, and the actual worsening of results following adaptation of our error mitigation is attributed to the rescaling of the 2σ occupation, which substantially affects CPs on the boundary of the NNA basin.

**Table 3:** Values of the electron density *ρ*(***r***) and its Laplacian ∇^2^*ρ*(***r***) at critical points in the density of a selection of molecules, comparing noise-free, noisy, and error mitigated data against a near-exact witness

Mol.	CP	*ρ*(***r***)				∇^2^*ρ*(***r***)			
		Noise-free	Noisy	Mitigated	Witness^a^	Noise-free	Noisy	Mitigated	Witness^a^
H_2_	Bond	0.2524	0.2506	0.2506	0.2684	−0.7818	−0.7542	−0.7542	−1.2445
LiH	Bond	0.0431	0.0399	0.0419	0.0394	0.1437	0.1177	0.1513	0.1553
Li_2_	NNA	0.0158	0.0134	0.0118	0.0129	−0.0139	−0.0102	−0.0085	−0.0143
Li_2_	Bond	0.0154	0.0132	0.0116	0.0121	0.0017	−0.0025	−0.0005	0.0101
Li_2_	Bond	0.0154	0.0132	0.0116	0.0121	0.0022	−0.0019	−0.0001	0.0101
H–CN	Bond	0.3934	0.3934	0.3934	0.4831	1.3149	1.2480	1.2385	−0.3586
HC-N	Bond	0.2635	0.2635	0.2635	0.2990	−0.7468	−0.7346	−0.7321	−1.3241

a CCSD/aug-cc-pVTZ level of theory.

Our examples serve to illustrate the need to account for noise imbalance in future NISQ-computation of chemistry that targets high fidelity and accuracy. What we mean by noise imbalance is that some observables are more protected against errors than others. For example, in the context of electron densities, symmetries of orbitals in the chosen active space, the encoding, the ansatz, *etc.*, can all affect relative noise when comparing local properties at different points in space. We note that multiple approaches exist that could potentially mitigate noise imbalance (see, for instance, ref. 57–60 and references therein).

## Methods

Calculations on H_2_ were performed with a hardware-efficient circuit on the Särimner device of Chalmers, employing 50 000 samples. Calculations for LiH were performed on ibmq_quito utilizing a hardware-efficient two-local ansatz using 8192 samples.^61^ To reduce the effects of noise, readout mitigation was applied to all hardware calculations. Calculations of H_2_ and LiH made use of parity encoding to reduce the required number of qubits and circuit depths.

Calculations of Li_2_ and HCN were performed on simulated devices using two consecutive layers of the ExcitationPreserving hardware-efficient ansatz available in Qiskit version 0.42.1.^61^ Simulations utilized a depolarizing noise model and were performed over 10^6^ samples. All calculations were additionally simulated without noise for comparison.

Our near-exact witness data was obtained with conventional quantum chemistry methods, using PySCF^62^ at the CCSD/aug-cc-pVTZ level of theory. Topological analyses of electron densities were performed with Critic2.^63^ Additional computational and hardware details are provided in the ESI.†

## Conclusions

Whereas tests of quantum computational accuracy are straightforward for small computational problems, for which conventional computation can be referenced, this will not always be so. Due to the rapid advances in quantum computational hardware, we may eventually face situations when it is not easy to validate whether a given problem has been solved to our satisfaction. The motivation behind this work is a proof-of-concept for utilizing electron densities as fidelity witnesses, future-proof benchmarks, for the quality of the quantum computation of materials and molecules. To that end, we have demonstrated the first topological analyses of electron densities inside molecules carried out with the assistance of quantum computers. Our work focuses on measuring one-particle reduced density matrices on real and simulated quantum hardware, how error mitigation can be applied to these entities, and how the resulting electron densities differ from near-exact reference calculations. The molecular systems studied herein are small enough to be treatable by current quantum hardware and small enough for comparisons with high-quality electron densities derived from conventional computations. Our examples are chosen to demonstrate the sometimes-detrimental effects of noise being imbalanced both spatially and with respect to 1-RDM entries. Therefore, we suggest that noise imbalance should be considered when designing or selecting error mitigation techniques, ansätze, encoding, and active spaces. Our work emphasizes the growing potential and need for high-quality (quantum) crystallography experiments,^64,65^*i.e.*, experimental determination of electron densities near the diffraction limit.

## Data availability

ESI† document provides details on quantum hardware, quantum simulations, noise model, quantum chemistry calculations, and additional results. Supplementary datafiles, including electron densities, 1-RDMs, and results of topological analyses are available at the Swedish National Data Service: http://doi.org/10.5878/0n2y-dp56.

## Author contributions

Conceptualization: MR, investigation (theoretical): MS, PL, WD, MR, investigation (experimental): CW, GT, JBy, device fabrication: JBi, AO, writing: MR, MS, WD, PL.

## Conflicts of interest

The authors report no conflicts of interest.

## Supplementary Material

SC-015-D3SC05269A-s001
