# Supplementary material for: The electron density: a fidelity witness for quantum computation
Source: Chem Sci. 2023 Dec 20;15(6):2257–65. doi: 10.1039/d3sc05269a (PMC10848700; doi:10.1039/d3sc05269a)
Supplement: SC-015-D3SC05269A-s001 [file SC-015-D3SC05269A-s001.pdf]

Electronic Supplementary Material (ESI) for Chemical Science.
This journal is © The Royal Society of Chemistry 2024

Supporting information to:

The Electron Density: A Fidelity Witness for Quantum Computation

1. Mårten Skogh[1,2], Phalgun Lolur[1], Werner Dobrautz[1], Christopher Warren[3], Janka Biznárová[3], Amr Osman[3], Giovanna Tancredi[3], Jonas Bylander[3], and Martin Rahm*[1]
2. Department of Chemistry and Chemical Engineering, Chalmers University of Technology, Gothenburg, Sweden
3. Data Science & Modelling, Pharmaceutical Science, R&D, AstraZeneca, Gothenburg, Sweden
4. Department of Microtechnology and Nanoscience MC2, Chalmers University of Technology, Gothenburg, Sweden

*Corresponding author: martin.rahm@chalmers.se

Table of Contents

CALCULATION DETAILS 2
HARDWARE CALCULATIONS 2
SIMULATED QUANTUM CALCULATIONS 2
CONVENTIONAL QUANTUM CHEMISTRY CALCULATIONS 2
TOPOLOGICAL ANALYSIS 2
HARDWARE DETAILS 3
SIMULATED NOISE MODEL 3
ESTIMATING SAMPLING UNCERTAINTY 3
ADDITIONAL DATA 4
ONE-PARTICLE REDUCED DENSITY MATRICES (1-RDM) 4
NATURAL ORBITAL OCCUPATIONS 4
MOLECULAR ORBITALS 5
ADDITIONAL Li_2 AND HCN DENSITY DIFFERENCE PLOTS 7
GRID BASED ELECTRON INTEGRATION 8
REFERENCES 9

Data Availability

All data used throughout this work is available through the following link:
https://doi.org/10.5878/0n2y-dp56

Calculation Details

Hardware Calculations

Qiskit[1] was used throughout this work to generate all problem Hamiltonians, as well as to map the fermionic states onto qubits. For H_2 and LiH, parity encoding was used, which allowed two-qubit tapering to reduce the total number of qubits in the calculations. Using a minimal STO-3G basis, the resulting states comprise 2 and 4 qubits, respectively. The LiH active space was reduced by freezing the Li 1s core orbital and removing the $2p_y$ and $2p_z$ orbitals (the p orbitals perpendicular to the bond axis). This removal of orbitals is motivated by the work of Kandala *et al.*[2] To increase the accuracy of our hardware calculations, we implemented hardware-efficient circuits. Figures S1 and S2 show the full circuits used for H_2 and LiH, respectively. Additionally, the two calculations were implemented on two different quantum computers. The H_2 problem on Chalmers' three-qubit Särimner device, and the LiH problem on IBM's five-qubit ibmq_quito chip.

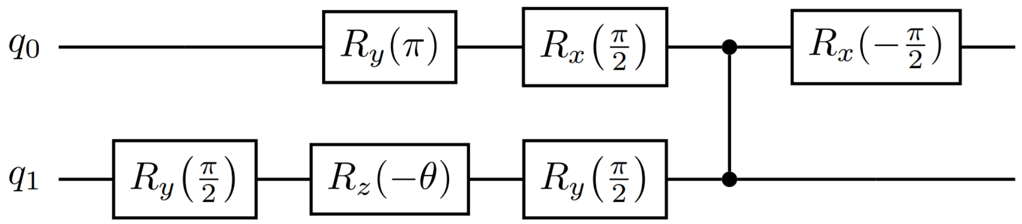

Figure S1: Hardware-efficient circuit used in the H_2 calculation.

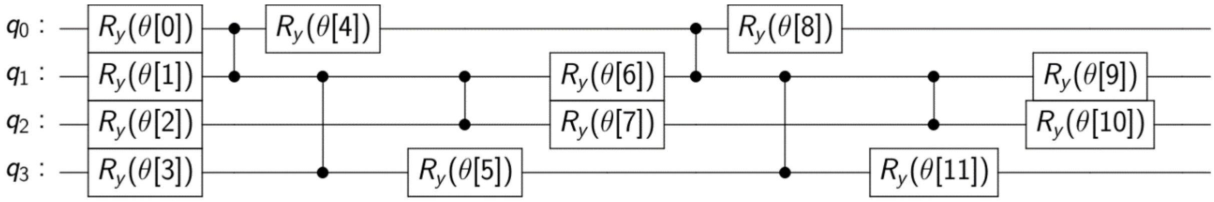

Figure S2: Hardware-efficient circuit used in the LiH calculation.

Simulated Quantum Calculations

Both our noisy simulations, for Li_2 and HCN, were implemented in Qiskit using a double-layer excitation preserving ansatz. A depolarizing noise model, including one- and two-qubit errors, was used to introduce noise effects, details of which are provided below. Due to technical issues with Qiskit, we could not utilize parity encoding for Li_2 and HCN and instead opted for a Jordan-Wigner encoding in these simulations. Noise-free statevector simulations were performed for all systems, including H_2 and LiH, to provide reference values and estimate the effects of noise. Both noise-free and noisy simulations used the COBYLA[3] optimizer during the VQE convergence.

Conventional Quantum Chemistry Calculations

In addition to our quantum computer calculations, we calculate our witness densities using PySCF, a Python package for conventional quantum chemistry[4]. These calculations are performed at the CCSD/aug-cc-pVTZ level of theory.

Topological Analysis

From the 1-RDMs calculated by either quantum computation, simulation, or conventional calculation, a cube file (Gaussian cube file format) was generated using PySCF. The cube files were subsequently used for the topological analysis with the software Critic2.[5]

Hardware Details

The Särimner device used for H_2 calculations is a three-qubit chip. Only two of the three qubits were used in encoding the H_2 state. The qubits are superconducting transmon qubits, and all three qubits are coupled through a single tunable coupler. Measured hardware characteristics are given in Table S1.

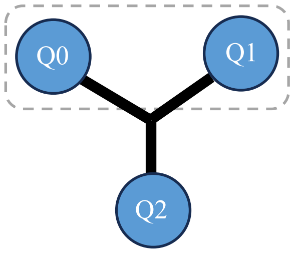

Figure S4: Coupling map for qubits Q0, Q1, and Q2 on Chalmers' Särimner quantum processor. The qubits within the dashed area were used for the calculation of H_2.

The ibmq_quito device is also a superconducting transmon qubit based processor that hosts five qubits, of which four were used in our calculations of LiH (Q0, Q1, Q2, Q3). The chip has a T-shaped coupling map, illustrated in Figure S3.

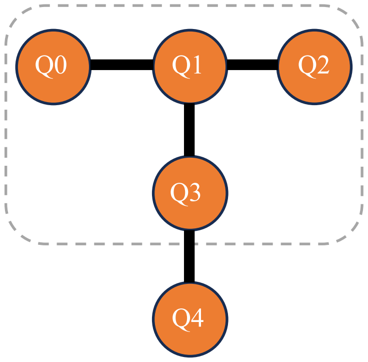

Figure S3: Coupling map for qubits Q0 through Q4 on the ibmq_quito. The qubits within the dashed area were used for the calculation of LiH.

Further details for both quantum processors can be found in the supporting information of Ref.[6]

Simulated Noise Model

To incorporate noise in our quantum hardware simulations, we implement a depolarizing noise model. The model applies random one- and two-qubit gate errors with probabilities 0.001 and 0.002, respectively. One can view the depolarizing channel as random, unintentional applications of additional gates after each intended gate in a circuit. The one-qubit depolarizing channel applies a random single-qubit gate, commonly one of the Pauli gates $\{\mathrm{X}, \mathrm{Y}, \mathrm{Z}\}$, with probability p_1 after each gate in the circuit. Similarly, for two-qubit errors, a two-qubit gate is chosen randomly from a set of Pauli products $\{\mathrm{IX}, \mathrm{IY}, \dots, \mathrm{ZY}, \mathrm{ZZ}\}$ with probability p_2. While the depolarizing model is computationally efficient, it should be noted that it gives a simplified description pf the noise processes in a physical quantum computer. More elaborate models exist, they however require more classical resources to simulate.

Estimating Sampling Uncertainty

For our hardware calculations, we have estimated the sampling uncertainty by providing upper and lower bounds equal to the standard deviation of the measured mean values. For the simulated calculations, this standard deviation is provided by Qiskit as part of the measurement procedure. For our calculations on physical hardware, we have estimated the standard deviation of the mean, s, as that of a Bernoulli distributed variable, $s_i(\bar{X}_i) = \sqrt{\frac{q_i p_i}{n_i}}$. Here, we define p_i as the probability of measuring the +1 eigenvalue and complementary $q_i = 1 - p_i$ as the

probability of measuring the -1 eigenvalue. We also use n_i to denote the number of samples used for measurement. For an operator $\hat{O}$ written as a sum of Pauli operators $\hat{P}_k$, the effective standard deviation can be calculated as $s(\langle\hat{O}\rangle)^2 = \sum_k s(\langle\hat{P}_k\rangle)^2$.

Additional Data

In this section, we present additional data and results from our calculations for the interested reader.

One-Particle Reduced Density Matrices (1-RDM)

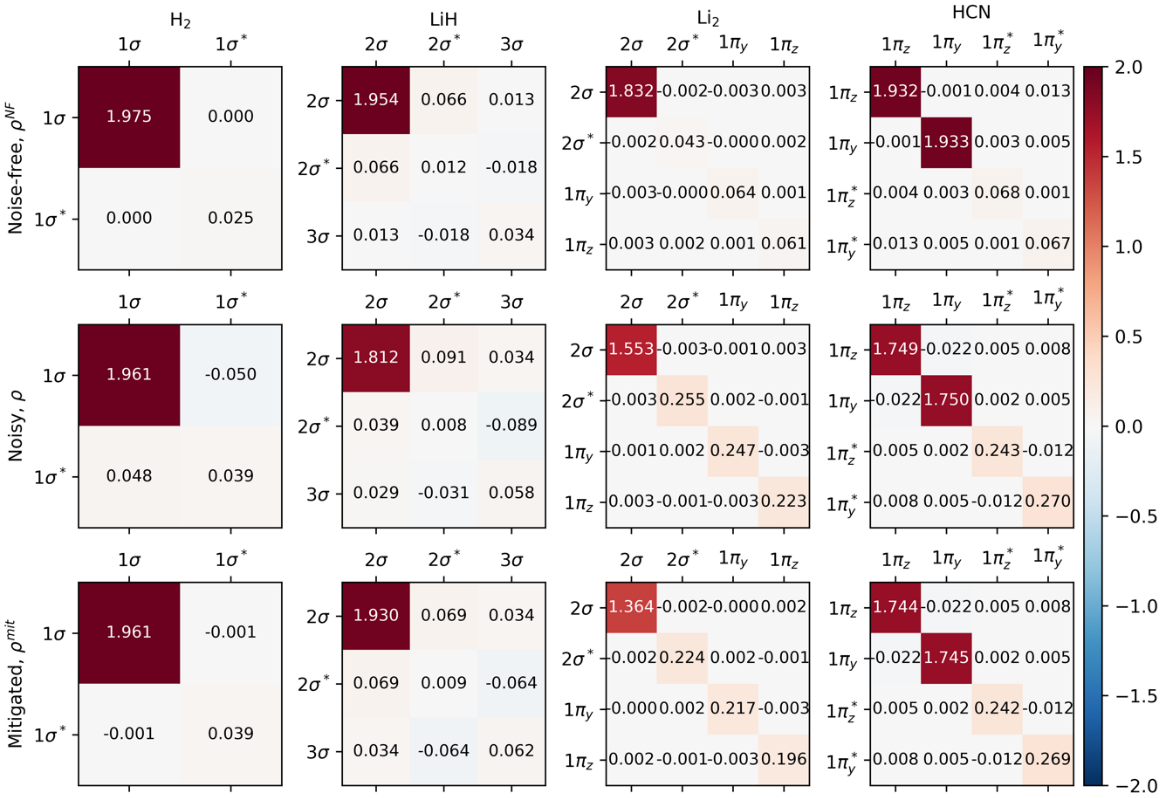

Figure S4: 1-RDMs of all non-witness calculations.

Natural Orbital Occupations

We calculate the natural orbital occupation by diagonalizing the 1-RDMs of the active spaces presented in Figure S4. The orbitals outside these active spaces correspond to Hartree-Fock orbitals, which are either fully occupied (2.0) or empty (0.0). The orbitals are then sorted, and the occupations are listed in Tables S1, S2, S3, and S4 for H_2, LiH, Li_2, and HCN, respectively, and the active space orbitals are indicated in orange.

Table S1: Natural orbital occupation after diagonalizing the H_2 1-RDMs in Figure S4. Active space orbitals are indicated in orange.

	Occupation		
Orbital #	Noise-free	Noisy	Mitigated
1	1.975	1.960	1.961
2	0.025	0.040	0.039

Table S2: Natural orbital occupation after diagonalizing the LiH 1-RDMs in Figure S4. Note the negative occupation of orbital 4 due to noise. Active space orbitals are indicated in orange.

	Occupation		
Orbital #	Noise-free	Noisy	Mitigated
1	2.000	2.000	2.000

2	1.956	1.814	1.933
3	0.044	0.092	0.104
4	0.000	-0.028	-0.038
5	0.000	0.000	0.000
6	0.000	0.000	0.000

Table S3: Natural orbital occupation after diagonalizing the Li_2 1-RDMs in Figure S4. Active space orbitals are indicated in orange.

	Occupation		
Orbital #	Noise-free	Noisy	Mitigated
1	2.000	2.000	2.000
2	2.000	2.000	2.000
3	1.832	1.553	1.364
4	0.064	0.255	0.224
5	0.061	0.247	0.217
6	0.043	0.222	0.195
7	0.000	0.000	0.000
8	0.000	0.000	0.000
9	0.000	0.000	0.000
10	0.000	0.000	0.000

Table S4: Natural orbital occupation after diagonalizing the HCN 1-RDMs in Figure S4. Active space orbitals are indicated in orange.

	Occupation		
Orbital #	Noise-free	Noisy	Mitigated
1	2.000	2.000	2.000
2	2.000	2.000	2.000
3	2.000	2.000	2.000
4	2.000	2.000	2.000
5	2.000	2.000	2.000
6	1.933	1.772	1.766
7	1.931	1.727	1.723
8	0.068	0.275	0.274
9	0.067	0.238	0.237
10	0.000	0.000	0.000
11	0.000	0.000	0.000

Molecular Orbitals

The orbitals used in the active spaces for H_2, LiH, Li_2, and HCN are presented in Figures S5, S6, S7, and S8, respectively. All bonds are aligned along the x-axis.

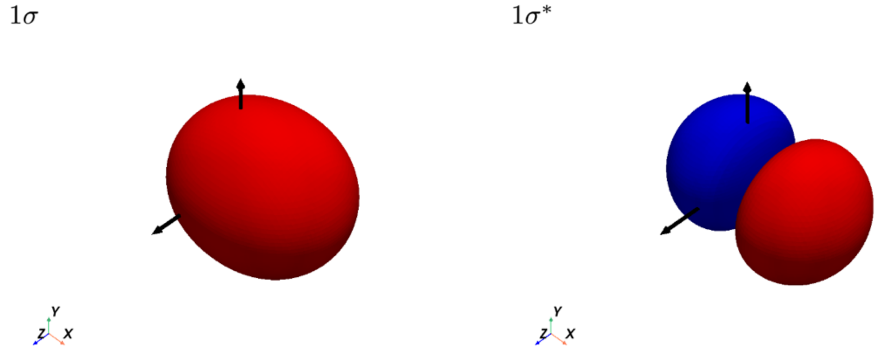

Figure S5: Canonical molecular orbitals in the active space for H2. Iso surfaces are shown for the values ±0.05.

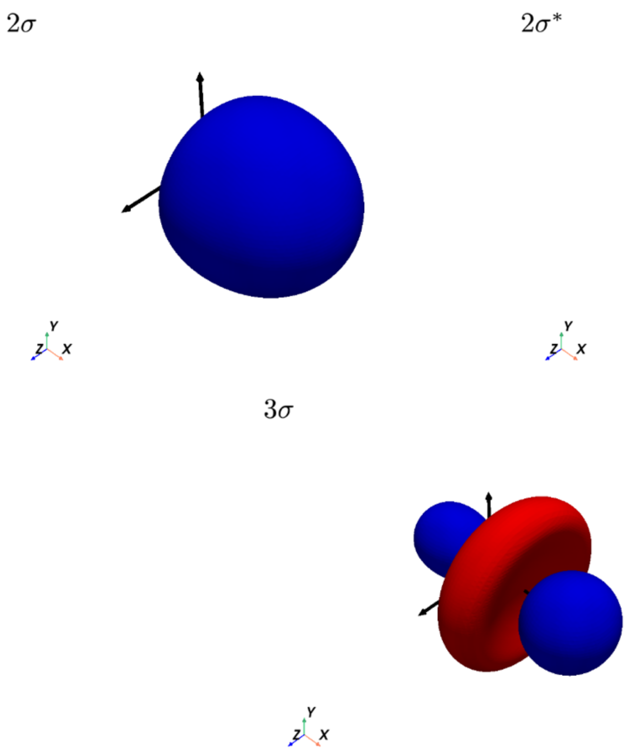

Figure S6: Canonical molecular orbitals in the active space for LiH. Iso surfaces are shown for the values ±0.05.

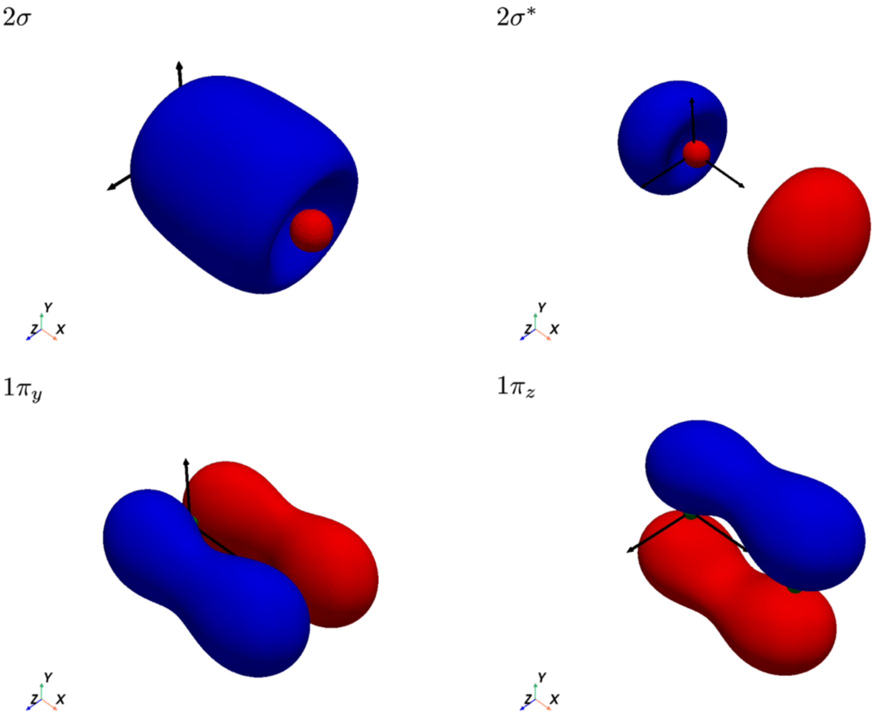

Figure S7: Canonical molecular orbitals in the active space for Li_2. Iso surfaces are shown for the values ±0.05.

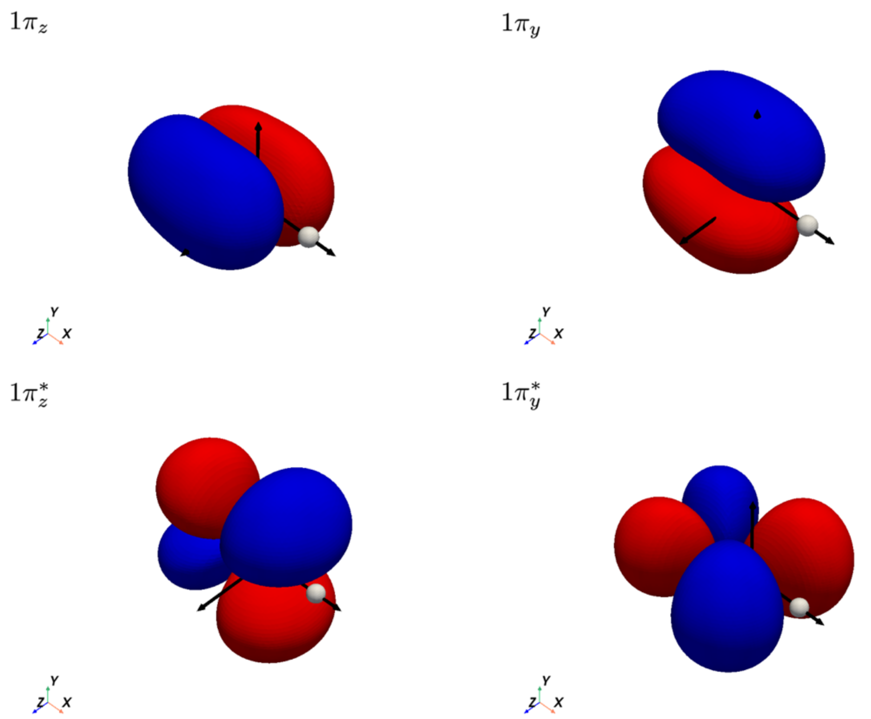

Figure S8: Canonical molecular orbitals in the active space for HCN. Iso surfaces are shown for the values ±0.05.

Additional Li_2 and HCN Density Difference Plots

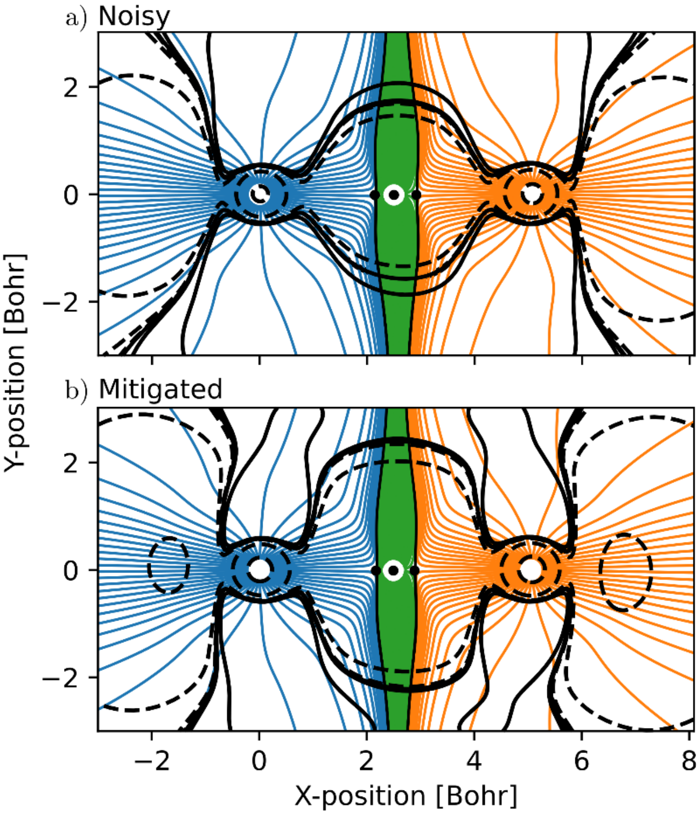

Figure S9: Topological analysis of Li_2. Contour plot compared to noise-free calculations for noisy (a) and mitigated (b) simulations. The contours are overlain streamlines showing the atomic basins for the Li_2 nuclei and NNA. Solid lines indicate a higher electron density when compared to the noise-free results; dashed lines indicate

a lower electron density. The contour lines are separated on a logarithmic scale. Lithium basins are shown in blue and orange, the NNA basin is indicated by green.

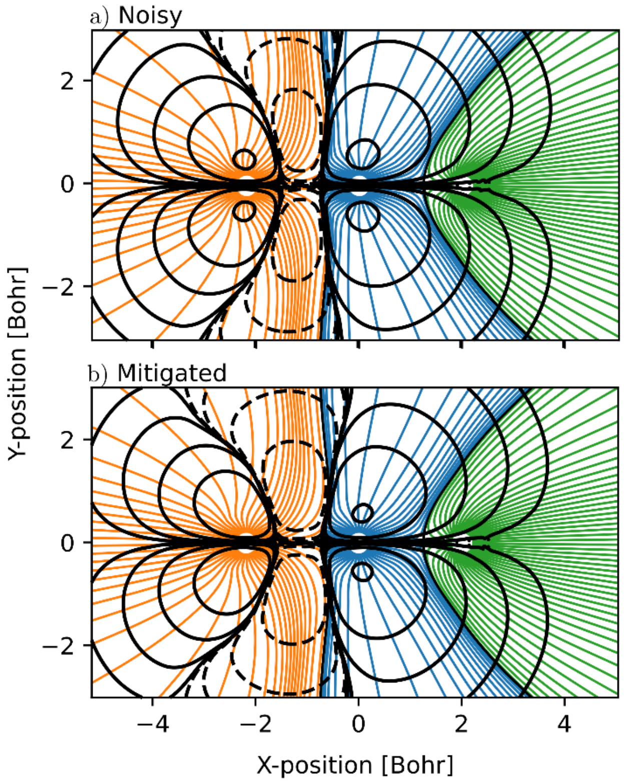

Figure S10: Topological analysis of HCN. Contour plot compared to noise-free calculations for noisy (a) and mitigated (b) simulations. The contours are overlain streamlines showing the atomic basins for the HCN nuclei. Solid lines indicate a higher electron density when compared to the noise-free results; dashed lines indicate a lower electron density. The contour lines are separated on a logarithmic scale. The basins of N, C, and H are indicated by orange, blue, and green, respectively.

Grid-Based Electron Integration

To validate our cube files, the integrated number of electrons was calculated for a large volume. The integrated value is in good agreement with the value derived from the trace of the 1-RDM (Table S1).

Table S5: Total electron numbers for the tested systems calculated using summation density integration and the trace of the 1-RDM.

Molecule	Integrated density[d]			Trace of 1-RDM		
H_2[a]	2.00	2.00	2.00	2.00	2.00	2.00
LiH[b]	4.00	3.88	4.00	4.00	3.88	4.00
Li_2[c]	6.00	6.28	6.00	6.00	6.28	6.00
HCN[c]	14.00	14.01	14.00	14.00	14.01	14.00

[a] Chalmers Särimner device. [b] ibmq_quito device. [c] Simulation using a depolarizing noise model. [d] A margin of 10 a.u. from the nearest nuclei was used when constructing the cube file.

References

1. Qiskit contributors. Qiskit: An Open-source Framework for Quantum Computing. Preprint at https://doi.org/10.5281/zenodo.2573505 (2023).
2. Kandala, A. *et al.* Hardware-efficient variational quantum eigensolver for small molecules and quantum magnets. *Nature* **549**, 242–246 (2017).
3. Powell, M. J. D. A Direct Search Optimization Method That Models the Objective and Constraint Functions by Linear Interpolation. in *Advances in Optimization and Numerical Analysis* 51–67 (Springer Netherlands, 1994). doi:10.1007/978-94-015-8330-5_4.
4. Sun, Q. *et al.* PySCF: the Python-based simulations of chemistry framework. *WIREs Computational Molecular Science* **8**, (2018).
5. Otero-de-la-Roza, A., Johnson, E. R. & Luaña, V. Critic2: A program for real-space analysis of quantum chemical interactions in solids. *Comput Phys Commun* **185**, 1007–1018 (2014).
6. Lolur, P. *et al.* Reference-State Error Mitigation: A Strategy for High Accuracy Quantum Computation of Chemistry. *J Chem Theory Comput* **19**, 783–789 (2023).
